# Safety in training for ultrasound guided internal jugular vein CVC placement: a propensity score analysis

**DOI:** 10.1186/s12871-021-01460-0

**Published:** 2021-10-08

**Authors:** Alessandro De Cassai, Federico Geraldini, Laura Pasin, Annalisa Boscolo, Francesco Zarantonello, Martina Tocco, Chiara Pretto, Matteo Perona, Michele Carron, Paolo Navalesi

**Affiliations:** 1 UOC Anaesthesia and Intensive Care Unit, University Hospital of Padua, 13, Gallucci St., 35121 Padua, Italy; 2 UOC Anaesthesia and Intensive Care Unit, Department of Medicine-DIMED, University of Padua, Padua, Italy

**Keywords:** Education, Training, Residents, CVC, Cohort study, Propensity score

## Abstract

**Background:**

Central venous catheter (CVC) placement is a routine procedure but is potentially associated with severe complications. Relatively small studies investigated if the use of ultrasound is effective in bridging the skill gap between proficient and not proficient operators, while patient safety during training remains a controversial topic. The first aim of this study was to evaluate if resident proficiency affects the failure rate in CVC positioning under ultrasound guidance. In addition, it aimed to investigate the different rate of complications between proficient and non proficient residents.

**Methods:**

We conducted a cohort study including CVC placed by residents at the University Hospital of Padova, from November 1, 2012 to July 9, 2020 comparing proficient and non proficient residents. To avoid bias the two cohorts were matched using propensity score.

**Results:**

A total of 356 residents positioned 2310 CVC during the 8 year study period. Among them, two groups of 1060 CVCs each were matched with a propensity score analysis. There was no difference in the failure rate among the groups (2.8 vs 2.7%, *p*-value 0.895).

Moreover, cohorts had the same rate of hematomas, catheter tip malposition, arterial puncture and pneumothorax. No cases of hemothorax were reported.

**Conclusions:**

We found the same rate of success and incidence of adverse complications among cohorts, meaning that the process of skill acquisition is safe as long as appropriate training and direct supervision by a senior consultant are available.

## Introduction

The placement of a central venous catheter (CVC) is considered a routine procedure in both the intensive care ward and the operating room. Despite this, it is potentially associated with severe complications such as arterial puncture or cannulation, hematoma, pneumothorax and hemothorax [1, 2]. Catheter insertion was traditionally carried out with a landmark based technique, though it has been gradually superseded by an ultrasound guided approach.

The use of ultrasound has been associated with fewer complications and is strongly recommended by current guidelines for internal jugular vein cannulation [3]. Previous studies have revealed that the use of ultrasound significantly reduces complication rate among not proficient operators (NPO) [4, 5].

Nonetheless, relatively small studies investigated if the use of ultrasound is effective in bridging the skill gap between proficient operator (PO) and NPO, with no definitive consensus ascertaining if there is a difference in complications between the two cohorts [6, 7]. While hands-on training is essential for NPO to become skilled in performing the procedure, patient safety must remain the priority. Therefore, we designed this study to provide a clear quantification of the risk gap between PO and NPO.

The primary objective of this study was to evaluate if resident proficiency affects the failure rate in CVC positioning under ultrasound guidance and direct consultant supervision. Secondary objectives were rate of procedure complication (arterial puncture, hematoma formation at the puncture site, pneumothorax, hemothorax and CVC malposition).

## Methods

A retrospective cohort study was conducted and the study protocol was in accordance with the 1964 Declaration of Helsinki and its later amendments. Study was approved by the Ethics Committee for Clinical Research of the Padova University Hospital (Chairman: Dr. Sergi; reference number 4961/AO/20). Informed consent was waived by the local IRB (Ethics Committee for Clinical Research of the Padova University Hospital).

A retrospective review of the records of all patients requiring a CVC admitted to the procedure room of the University Hospital of Padova, from November 1, 2012 to July,92,020, was performed. All ultrasound-guided internal jugular vein (IJV) cannulation with out-of-plane technique performed in the procedure room of our institution were included in the study. Exclusion criteria were: patients’ age < 18 years old, insertion site other than IJV, technique other than out-of-plane and use of landmark technique.

From the first year of residency residents follow frontal lessons (e.g. anatomy, CVC usage and related complications) and attend operating rooms in order to be familiar with surgical patients, devices and by using ultrasound in different settings (e.g locoregional anesthesia). Moreover, at our institution (University Hospital of Padova) since 2007 there is an active simulation center (https://www.simularti.it/) with a variety of skill trainers available and different simulation courses. Since the first year residents are involved in these courses in order to develop both ultrasound and eye-hand coordination through practice.

In the procedure room of our institution CVC are positioned by residents from the first to last year always under the direct supervision of a consultant. The supervisor remains a passive observer during the CVC placement, intervening only in cases of inappropriate procedure execution or request by the resident. Intervention may span from verbal correction and tips up to hand-on assistance, depending on the situation and expertise of the resident performing the procedure.

**Fig. 1:**
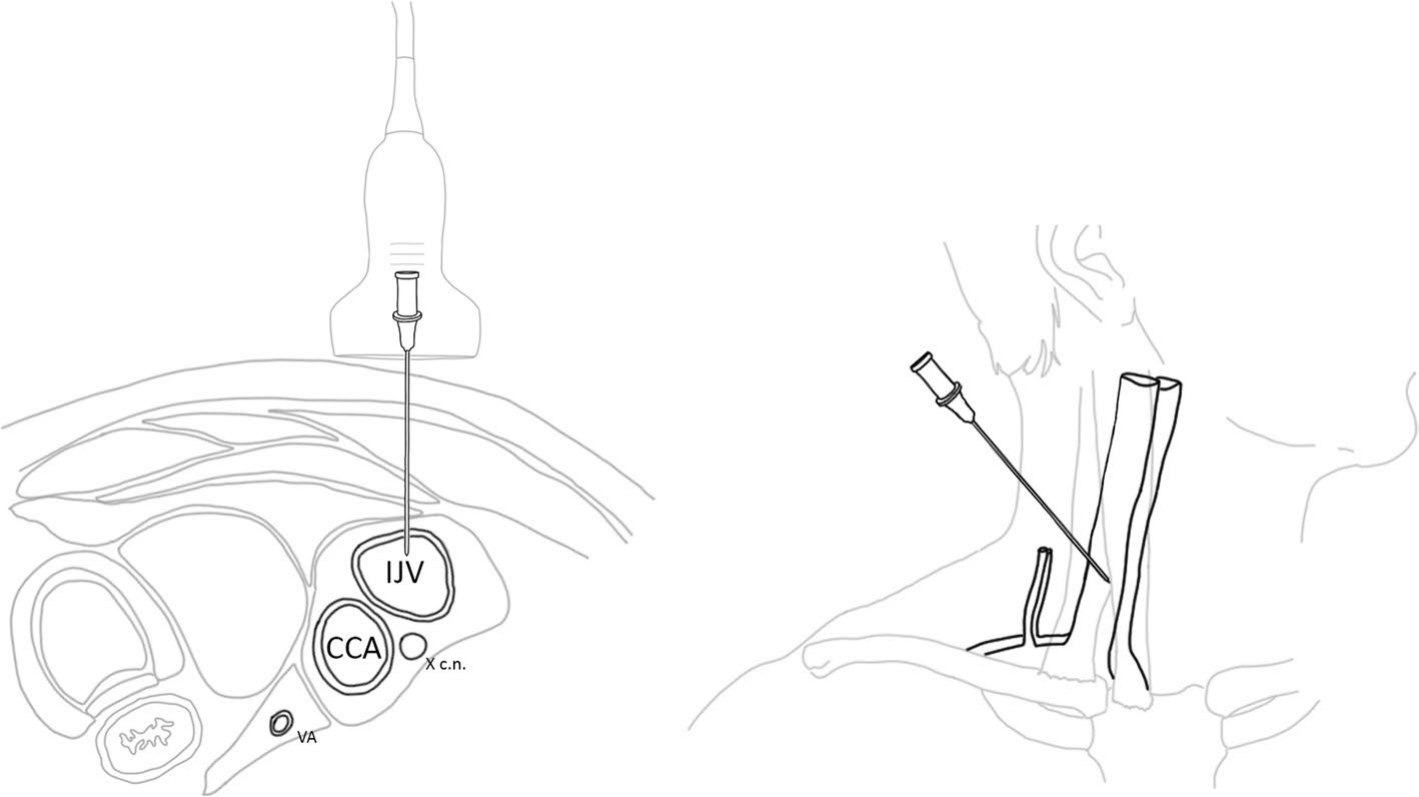
Out of plane technique

The routine technique for CVC placement at our institution is the out-of-plane technique: the probe is kept in a transverse position relative to the vessel while the needle is advanced following an out-of-plane view (Fig. 1). After catheter insertion all the data relative to the procedure (number of operators, technique and complications) are reported on a specific register.

We divided the CVC positioning procedures in two groups according to the operator’s proficiency (NPO group and PO group).

Proficiency was defined acquired during the residency on case by case evaluation as per local institution protocol, usually it is ‘acquired’ when last year residents performed at least 50 or more catheterizations [8].

Primary outcome was the rate of procedure failure, defined as the need for hands-on assistance of another operator during the procedure. Secondary outcome was the rate of procedure complication (arterial puncture, hematoma formation at the puncture site, pneumothorax, hemothorax and CVC malposition) in NPO and PO groups.

Needs for hands-on assistance, arterial puncture and hematoma formation were evaluated through inspection of the procedure room register.

Hemothorax, pneumothorax and incorrect CVC position were evaluated through inspection of the post-procedure chest radiography and the CVC was defined as malpositioned if the tip of the catheter was placed outside veins or in subclavian, axillary or contralateral jugular vein. Arterial puncture rate was retrieved from procedure chart while hematoma formation was evaluated from both procedure chart and patient diary for the first 24 h post-procedure.

To avoid bias arising from patient and catheter characteristics the following patients’ data were collected: age (years), gender (M/F), CVC diameter (Fr), site of insertion and laboratory tests (Prothrombin Time (PT), Partial Thromboplastin Time (PTT) and platelets count).

### Statistical analysis

To calculate an adequate sample size for the matched analysis, we considered 4% as the incidence of failure rate [9]. We aimed to show a doubling incidence in the NPO group resulting in at least 739 couples (1478 total matched patients) with a power of 90% and a significance level of 0.05.

Data for each continuous variable was analyzed for a normal distribution using the Shapiro-Wilk test. Results for continuous variables with normal distributions were expressed as mean and standard deviation values; those with non-normal distributions were expressed as median and first and third quartile values. Analysis of data with a normal or a non-normal distribution was performed using the two-tails Student’s t-test and the Mann-Whitney U test, respectively.

The results for analyses of categorical variables were reported as percentages and were compared between groups using the Chi-square test or Fisher’s exact test as appropriate.

To neutralize the effect of confounding variables a propensity score matching analysis was performed using the nearest method with a 0.05 caliper [10] on clinically significant confounder. We defined the following patient related parameters as confounders: age, gender, site of insertion, catheter diameter, PT, PTT and platelets count.

The balance among the baseline cohorts and the final balance of the matched cohort was assessed using the standardized differences, with less than 0.05 chosen to indicate adequate balance.

A subgroup analysis was conducted analyzing the PO group with a neophyte operators (NO) subgroup defined as operators with less than 2 years of experience and who performed 20 or less catheterizations.

*P*-values ≤0.05 were considered statistically significant. All statistical analyses were performed using R version 4.0.2 (2020-06-22).

## Results

A total of 356 residents positioned 2310 CVC during the 8 year study period. Among them, two groups of 1060 CVCs each were matched with a propensity score analysis, and standardized differences showed a balance among the matched cohorts.

Twenty-two residents (6.2%) were in the PO group at the beginning of the study, while 104 (29.2%) were in the NPO group at the end of the study. All the remaining 230 (64.6%) residents initially in the NPO group progressed to PO group as they reached the proficiency criteria.

Mean patients’ age in our cohort was 70.3 ± 15.88 years and 48.9% were female. A total of 1156 procedures were carried out by PO, the remaining 1154 were performed by NPO. Most procedures (78.1%,) were right side cannulations.

There was no difference in the failure rate among the groups (2.8 vs 2.7%, *p*-value 0.895).

Moreover, NPO and PO had the same rate of hematomas (1.7 vs 1.6%, *p*-value 0.864), catheter tip malposition (2.6 vs 2.1%, *p*-value 0.390), arterial puncture (1.6 vs 1.6%, *p*-value 1) and pneumothorax (0.9 vs 0.4%, *p*-value 0.108). No cases of hemothorax were reported (Table 1).

**Table 1:** Cohort characteristics and outcomes

	Overall Population			Propensity score			StD
	NPO (n:1156)	PO (n:1154)	*p*-value	NPO (n: 1060)	PO (n: 1060)	*p*-value	
Age	70.40 ± 15.85	70.22 ± 15.91	0.787	70.80 ± 15.70	70.52 ± 15.98	0.686	0.02
Sex (F)	50.3 (581)	47.4 (547)	0.169	50.0% (530)	51.1% (542)	0.602	0.02
Site (sin) (%)	21.3% (246)	22.5% (260)	0.467	22.2% (235)	22.5% (238)	0.834	0.01
Catheter							
7 Fr	72.6% (839)	69.2% (799)	0.125	72.3% (766)	71.8% (761)	0.957	0.01
8 Fr	15.7% (182)	16.46% (190)		16.4% (174)	16.9% (179)		
12 Fr	11.7% (135)	14.3% (165)		11.3% (120)	11.3% (120)		
Laboratory findings							
PTT(s)	29.25 ± 7.94	29.46 + −9.47	0.547	28.95 ± 6.97	29.14 ± 8.19	0.564	0.02
PT(%)	68.99 ± 20.71	69.47 + − 27.10	0.632	69.40 ± 20.43	69.31 ± 27.63	0.933	0.01
PLT (10^9^/L)	208.65 ± 134.21	206.75 ± 131.23	0.730	210.19 ± 128.32	206.56 ± 131.84	0.521	0.03
Outcomes							
Failure	3.0% (35)	2.5% (29)	0.451	2.8% (30)	2.7% (29)	0.895	
Hematoma	1.9% (22)	1.5% (17)	0.422	1.7% (18)	1.6% (17)	0.864	
Malposition	2.5% (29)	2.0% (23)	0.403	2.6% (28)	2.1% (22)	0.390	
Pneumothorax	1.0% (11)	0.3% (4)	0.071	0.9% (10)	0.4% (4)	0.108	
Artery puncture	1.7% (20)	1.6 (18)	0.747	1.6% (17)	1.6% (17)	1	

*PT* Prothrombin Time, *PTT* Partial Thromboplastin Time, *PLT* Platelet count, *StD* Standardized differences

Furthermore, subgroup analysis comparing PO with NO also reported no significant differences (Table 2).

**Table 2:** Subgroup analysis

	Overall Population			Propensity score		
	N (n:224)	PO (n:1154)	*p*-value	N (n:224)	PO (n: 224)	*p*-value
Age	70.26 ± 16.19	70.22 ± 15.91	0.973	70.26 ± 16.19	70.08 ± 16.84	0.906
Sex (F)	50.0% (112)	47.4% (547)	0.611	50.0% (112)	47.8%	0.636
Site (L)	17,4% (39)	22.5% (260)	0.085	17,4% (39)	17.0%	0.900
Catheter						
7 Fr	83.9% (188)	69.2% (799)	< 0.001	83.9% (188)	81.2% (182)	0.698
8 Fr	4.5% (10)	16.46% (190)		4.5% (10)	4.5% (10)	
12 Fr	11.6% (26)	14.3% (165)		11.6% (26)	14.2% (32)	
Laboratory findings						
PTT(s)	29.36 ± 7.03	29.46 ± 9.47	0.844	29.36 ± 7.03	28.64 ± 7.69	0.302
PT(%)	69.18 ± 21.95	69.47 ± 27.10	0.864	69.18 ± 21.95	67.90 ± 24.32	0.557
PLT (10^9^/L)	193.35 ± 123.53	206.75 ± 131.23	0.142	193.35 ± 123.53	206.35 ± 119.83	0.259
Outcomes						
Failure	1.8% (4)	2.5% (29)	0.424	1.8% (4)	2.2% (5)	0.736
Hematoma	1.8% (4)	1.5% (17)	0.726	1.8% (4)	2.2% (5)	0.736
Malposition	3.6% (8)	2.0% (23)	0.145	3.6% (8)	3.1% (7)	0.792
Pneumothorax	1.3% (3)	0.3% (4)	0.056	1.3% (3)	0.9% (2)	0.652
Artery puncture	0.4% (1)	1.6 (18)	0.191	0.4% (1)	2.7% (6)	0.056

*PT* Prothrombin Time, *PTT* Partial Thromboplastin Time, *PLT* Platelet count

## Discussion

Our study showed that there was no difference in either success rate and complication rate among NPO and PO when placing IJV with ultrasound guidance, implying that this procedure is safe even while acquiring proficiency. To the best of our knowledge, this is the largest study evaluating the skill-gap in ultrasound-guided CVC placement among PO and NPO.

Although CVC placement may lead to several complications, their incidence has dramatically decreased with the guidance of ultrasound [6, 11] and its use is strongly recommended in daily clinical practice [3].

Two previous studies investigated the effect of operator proficiency in ultrasound CVC placement [4, 5]. Mey et al. [5] investigated complication and success rate using a two operators technique (ultrasound and puncturing operator). In their study neither success rate nor complication rate was dependent on the experience of the puncturing physician, however it was highly dependent on the proficiency of the ultrasound operator. Technology in ultrasound machines and probes has undergone dramatic change since its inception, leading to images with high spatial and contrast resolution [11]. Therefore, it is not surprising that in Mey’s study [5], performed 20 years ago, the ultrasound operator’s proficiency was essential for the correct visualization of the anatomical structure and for the success of the procedure. Rando et al. [4] analyzed the skill gap among PO and NPO in mixed landmark and ultrasound assistance cohorts. While they found that the overall success rate was dependent on the operator’s proficiency, this was not statistically significant when only the subgroups using ultrasound were compared for both success (95 vs 86%) and complication rate (8.3 vs 7.8%). Even if these findings are similar to ours, given the low sample size of these subgroups it is not possible to infer additional conclusions.

In our study, complications were rare and in line with the available literature on ultrasound guided IJV catheter positioning (tip malposition 2.3%, hematoma formation 1.7%, artery puncture 1.6%, pneumothorax 0.6%, hemothorax 0%) [12]. Moreover, we found no significant difference in success and complication rate even when considering PO and NO, implying that the use of ultrasound with proper training and supervision could potentially render this procedure safe even when performed by operators with very limited experience. Though interesting, we believe the scarce numerosity limits the impact of these results.

Our study presents several limitations that need to be discussed. The first one is linked to study design (historical and single center study). Rate of some complications such as arterial puncture and hematoma could have been affected by a recording bias, due to the absence in the analyzed dataset of monitoring and charting tools specific for these complications.

Secondly, the absence of significance in our cohort may be related to an inadequate sample size for some complication with a very low incidence (i.e. pneumothorax).

Thirdly, we defined PO as operators within the last year of residency with at least 50 previous successful procedures. While this choice is supported by literature [4, 8] we recognize that this is an arbitrary cut-off.

Even if our study shows that CVC can be safely placed under ultrasound guidance by NPOs, it should be emphasized that before undertaking CVC placement, NPOs follow a strict education program since the first year of residency [13]. Therefore, the take-home message from our manuscript is far from endorsing and encouraging the practice of placing a CVC under ultrasound guidance without proper training and know-how. Moreover, we have to stress that for each CVC placed there was a consultant available for hints and suggestions, which, as shown by literature, is crucial and significantly affects success rate [14].

## Conclusions

The placement of an IJV catheter recorded the same success rate and was equally safe when performed by either PO and NPO as long as they have appropriate training and the direct supervision of a consultant. Multicentric prospective trials on larger samples are needed to confirm our results, especially to evaluate rare complications with a low incidence.

## Data Availability

The datasets generated and analysed during the current study are available from the corresponding author on reasonable request.

## Abbreviations

CVC: Central venous catheter
IJV: Internal jugular vein
NPO: Not proficient operators
PO: Proficient operator
PTT: Partial Thromboplastin Time
PT: Prothrombin Time
